# La lucite polymorphe: à propos d’un cas

**DOI:** 10.11604/pamj.2019.34.12.16212

**Published:** 2019-09-06

**Authors:** Jawad El-Azhari, Mohammed Boui

**Affiliations:** 1 Service de Dermatologie-Vénérologie, Hôpital Militaire d’Instruction Mohammed V, Rabat, Maroc

**Keywords:** Photodermatose, lucite polymorphe, antipaludéens de synthèse, Photodermatosis, polymorphous light eruption, synthetic antimalarial drugs

## Abstract

Polymorphic lucite (PL) is a frequent photodermatosis, but its pathophysiologic mechanism is still poorly elucidated. We report the case of a 54-year old male teacher, with no previous medical-surgical history, presenting with recurrent papular and very pruritic rash lasting for 12 years. Clinical examination showed papular-vesicular excoriated lesions on the face, the neck and the scalp. Eczematiform lesions occurred on the back of the hands. The remainder of the integument as well as the mucous membranes were spared. The suspected diagnoses were lupus, drug-induced photosensitization or contact photosensitivity and polymorphic lucite (PL). During the interview, the patient reported that this eruption had recurred in the same period every year, namely at the beginning of spring and lasted until the end of summer. Photobiological evaluations were not performed. Antinuclear antibody test was negative, and histological examination was non-specific showing dermal dense lymphocytic infiltrate. The diagnosis of LP was suspected and the patient underwent synthetic antimalarial drug-based therapy associated with external photoprotection.

## Image en médecine

La lucite polymorphe (LP) est une photodermatose fréquente, mais son mécanisme physiopathologique reste encore mal élucidé. Nous rapportons le cas d’un homme de 54 ans, instituteur de profession, sans antécédents notables, qui consulte pour une éruption papuleuse et très prurigineuse récidivante depuis une dizaine d’années. À l’examen il s’agit de lésions papulo-vésiculeuses excoriées intéressant le visage, le cou et le cuir chevelu. Les dos des mains sont le siège de lésions eczématiformes. Le reste du tégument ainsi que les muqueuses sont épargnés. Les diagnostics évoqués étaient un lupus, une photosensibilisation médicamenteuse ou de contact et une lucite polymorphe (LP). En reprenant l’interrogatoire, le patient rapporte que cette éruption récidive chaque année à la même période, à savoir le début du printemps et dure jusqu’à la fin de l’été. Les explorations photobiologiques n’ont pas été réalisées. Les anticorps anti-nucléaires étaient négatifs, et l’histologie non spécifique montrant cependant un infiltrat lymphocytaire dense dermique. Le diagnostic de LP a été posé, et le patient mis sous antipaludéens de synthèse associés à une photoprotection externe.

**Figure 1:**
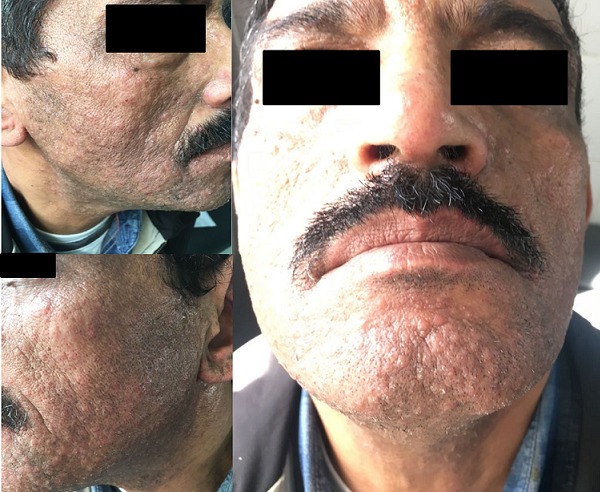
Lésions papulo-vésiculeuses excoriées intéressant le visage et le cou

